# Do Medical Students Suffer from Chronic Diseases? A Secondary Cross-Sectional Analysis of a Medical School in Portugal

**DOI:** 10.3390/ijerph22081282

**Published:** 2025-08-16

**Authors:** Filipe Prazeres, Diogo Maia, Marta Duarte

**Affiliations:** 1 Faculty of Health Sciences, University of Beira Interior, 6200-506 Covilhã, Portugal; a41014@fcsaude.ubi.pt (D.M.); martad@fcsaude.ubi.pt (M.D.); 2 Family Health Unit Beira Ria, 3830-596 Gafanha da Nazaré, Portugal; 3 RISE-Health, Department of Community Medicine, Information and Health Decision Sciences, Faculty of Medicine, University of Porto, 4200-450 Porto, Portugal

**Keywords:** chronic diseases, family history, medical students, Portugal

## Abstract

Background/Objectives: There is still debate about students’ health in medical schools. The aim of this study was to assess the proportion of chronic diseases among medical students and analyze their predictors. Methods: We performed a secondary cross-sectional analysis using a database from a single Portuguese university. The sociodemographic/clinical variables of 309 medical students were included. Logistic regression was performed to ascertain the effects of sex, age, medical course year, financial situation, and family history of chronic diseases on the likelihood of medical students having chronic disease. Results: Median age (Q1, Q3) was 21 (17, 43) years, with females comprising 79.9% of the sample. The distribution of students by school year was similar. The majority (65.7%) reported having sufficient money. Twenty-three percent suffered from chronic disease, and around forty percent had parents or siblings with chronic disease. In the regression model, only a family history of chronic disease was associated with a personal history of chronic disease. Medical students with parents or siblings who have a chronic disease are 3.3 times more likely to have a chronic disease themselves. Conclusions: Future interventions targeting the medical student population will be needed in Portugal to reduce the prevalence and burden of chronic diseases, particularly among those with a positive family history.

## 1. Introduction

There is still debate about whether attending medical school impacts students’ health [1]. Some authors found that while the majority of medical students remain healthy, there are exceptions who face specific health challenges regarding healthy lifestyle habits [1]. A sedentary lifestyle [2,3,4], unhealthy diet [2,3,4], alcohol drinking [4], tobacco smoking [4], and poor sleep quality [5] are common among medical students. This is important not only for the students’ health, as these are risk factors for chronic diseases (health conditions lasting 6 months or longer [6]), but also for their future patients, since healthier physicians are better able to counsel their patients regarding unhealthy habits [7,8].

In recent years, there has been a noticeable shift in how the medical community addresses the health and well-being of healthcare professionals. Physicians, medical educators, and educational institutions have increasingly recognized the importance of supporting doctors’ physical and mental health [9]. This growing awareness reflects a broader commitment to fostering healthier study and work environments and ensuring that future doctors are equipped to care not only for their patients but also for themselves.

While it has been noted that medical students often lack sufficient knowledge about the impact of lifestyle behaviors on health [10], it was found that during the COVID-19 pandemic, they were more concerned about health-promoting behaviors than non-medical students [11].

Portugal has several national programs to promote healthy lifestyles and prevent chronic diseases in the general population. One example is the recent publication of the Despacho no. 12634/2023, which mandates the implementation of an Integrated Care Model for the Prevention and Treatment of Obesity, focused on organizing health services and professionals, as well as specific actions for identifying, referring, and monitoring individuals with pre-obesity or obesity targeted by health promotion, disease prevention, early diagnosis, and intervention measures [12].

The aim of this study is to assess the proportion of chronic diseases among medical students and to analyze the available predictors of a personal history of chronic disease using an existing database from a single Portuguese university.

## 2. Materials and Methods

This study is a secondary cross-sectional analysis of data collected between October and November 2023 from a sample of 310 medical students from a single Portuguese university. The sample represents approximately one-third of the 1023 students enrolled in the medical course at the time of data collection (response rate of 30.3%). Assuming the most conservative scenario (a proportion of 50%), this sample yields a margin of error of ±4.65 percentage points at a 95% confidence level. Data were collected using an anonymous online questionnaire administered via Google Forms. The survey link was distributed through the institutional email system to all medical students. Participation was voluntary, and informed consent was obtained at the beginning of the questionnaire.

For this secondary analysis, we extracted sociodemographic and clinical variables for 309 medical students. One participant was excluded due to the non-disclosure of sex. The primary outcome variable was personal history of chronic disease, assessed through the following self-reported question: “Do you have any disease or health problem that has lasted for more than six months or is expected to last for more than six months?” (yes/no). No specific disease or condition was specified.

The independent variables included age (in years), sex, medical course year, financial situation, and family history of chronic diseases, with the latter assessed with the following question: “Do your parents or siblings have any disease or health problem that has lasted for more than six months or is expected to last for more than six months?” (yes/no). No specific disease or condition was specified.

Ethical approval for the original data collection was obtained from the Ethics Committee of the University of Beira Interior (CE-UBI-Pj-2023-053). All data were already de-identified in the original database prior to this secondary analysis.

Regarding data analysis, IBM SPSS Statistics (Version 21) was utilized. Descriptive and inferential analyses were conducted. A logistic regression was performed to ascertain the effects of sex, age, medical course year, financial situation, and family history of chronic diseases on the likelihood of medical students having chronic disease.

A cut-off value of *p* < 0.05 was used to determine whether the results were statistically significant.

## 3. Results

The median age (Q1 = first quartile, Q3 = third quartile) was 21 (17, 43) years, with females comprising 79.9% of the sample. The distribution of students by school year was similar. The majority (65.7%) reported having sufficient money. Less than a quarter of the sample (23%) suffered from chronic disease, and around 40% had parents or siblings with chronic disease (Table 1).

Table 2 shows that in the logistic regression model, only a family history of chronic disease is associated with a personal history of chronic disease. Medical students with parents or siblings who have a chronic disease are 3.3 times more likely to have a chronic disease themselves. No sociodemographic variables were significant.

## 4. Discussion

In the analyzed database, it was found that the majority of medical students are free from chronic diseases. However, a notable portion, 23%, did report having a chronic disease. This figure is lower than the national prevalence of 57.8% but aligns more closely with the prevalence observed in the 25–34 age group (25.7%) [13]. It was also found that family history of chronic disease is a predictor of personal history of chronic disease. From the literature, it is known that family members not only share a genetic susceptibility but also often share environmental factors, lifestyle habits, and behaviors due to living together or in close proximity [14]. These shared factors can contribute to the prevalence of chronic diseases within the family [14]. Other authors have found that at least three in ten medical students have a family history of hypertension or diabetes [3].

One of the main limitations of this study is that all participants in the database are from a single medical school, which limits the generalizability of the findings to similar institutions. The sample also includes a disproportionately high percentage of female participants (79.9%) compared to the overall population of medical students (72%) [15], which may further limit the representativeness of the results. Additionally, personal and family histories of chronic diseases were assessed based on students’ self-perceptions—an indirect method previously used in other studies—which may have resulted in under- or overestimation. Self-reported data may also be subject to social desirability bias. Also, the database lacks variables related to lifestyle habits that could help explain the prevalence of chronic diseases observed. Importantly, the database does not provide information on specific chronic diseases, which restricts the ability to perform disease-specific analyses or comparisons. As this is a secondary analysis, this study was constrained by the variables available in the original dataset, which limited our ability to include certain predictors commonly used in chronic disease research. Lastly, the cross-sectional design of the study prevents any causal inferences. To enhance generalizability and provide more comprehensive insights, future research should include larger, more diverse samples and incorporate additional relevant variables that were not available in the current dataset.

The present study’s findings are significant despite their limitations. First, the presence of chronic diseases in nearly one-quarter of a young, educated population is concerning and may reflect early-onset health issues or the impact of high academic stress typical of medical training. A recent study in Portugal revealed that six in ten higher education students feel physically exhausted, and four in ten use psychotropic medication (such as anxiolytics, antidepressants, or sedatives) at least once a week [16]. This study, which involved over 2300 students aged between 17 and 35 years from public and private institutions across the country [16], underscores the mental health challenges faced by students. This highlights the need for medical schools to take a more proactive approach to student health, particularly in terms of screening and preventive care.

Additionally, the observed relationship between family and personal history of chronic disease reinforces previously established evidence on the role of genetic and family risk factors [17,18], even in populations presumed to have higher health literacy. For example, in Korea, family history has been identified as a significant risk factor for developing chronic conditions such as hypertension, dyslipidemia, diabetes, and arthritis, even when controlling for gender, age, income status, education level, and residence [19]. Similarly, among Mexican university students, a family history of type 2 diabetes and high blood pressure has been found to increase the likelihood of being overweight or obese [20]. In India, a family history of cardiovascular disease was associated with higher body mass index (BMI) among first-year medical students [21], and in another sample of medical students, it was associated with hypertension and obesity [22]. These findings highlight the important role of family history in risk prediction and the potential for more personalized disease prevention strategies [23].

While the lower prevalence of chronic disease compared to the general population might initially appear reassuring, it still raises questions about potential underreporting due to self-perception biases or lack of disease awareness, especially given the indirect nature of data collection. Moreover, the absence of information on specific chronic conditions and lifestyle factors limits the depth of the analysis. Nonetheless, the findings highlight an important baseline: medical students are not immune to the burden of chronic disease. This calls for further, more detailed research to explore underlying causes and inform institutional policies that support student well-being.

Overall, this study offers a valuable starting point for addressing health challenges in future healthcare professionals. This is especially important given previous research indicating that individuals with a personal or family history of chronic disease often do not follow recommendations for healthier lifestyles, particularly with regard to obesity-related measures [24].

## 5. Conclusions

The results from this study show that nearly one-quarter of the sample has a chronic disease. Future interventions (e.g., promoting healthy lifestyles) targeting the medical student population will be needed in Portugal to reduce the prevalence and burden of chronic diseases, particularly among those with a positive family history. Given that Portugal performs well in most behavioral risk factors, except for obesity and physical inactivity [25], these interventions may include improving access to physical activity opportunities, providing nutrition support, and fostering a culture of wellness on campus. Encouraging medical students to adopt healthier lifestyles will not only benefit their own health but may also enhance the quality of care they provide to future patients [26].

## Figures and Tables

**Table 1:** Sociodemographic and health data (*n* = 309).

	*n* (%)
Sex, *n* (%)	
Male	62 (20.1)
Female	247 (79.9)
Age (years), mdn (Q1, Q3)	21 (17, 43)
Medical course year, *n* (%)	
1st year	57 (18.4)
2nd year	40 (12.9)
3rd year	58 (18.8)
4th year	56 (18.1)
5th year	36 (11.7)
6th year	62 (20.1)
Financial situation, *n* (%)	
Not enough money	21 (6.8)
Sufficient money	203 (65.7)
Surplus money	85 (27.5)
Personal history of chronic disease, *n* (%)	
Yes	71 (23.0)
No	238 (77.0)
Family history (parents/siblings) of chronic disease, *n* (%)	
Yes	124 (40.1)
No	185 (59.9)

**Table 2:** Logistic regression model of predictors of personal history of chronic disease.

Characteristic	OR (95% CI)	*p* Value
Sex		
Male	base	
Female	0.9 (0.5 to 1.8)	0.801
Age	1.0 (0.9 to 1.1)	0.848
Medical course year		
1st year	base	
2nd year	0.8 (0.3 to 2.2)	0.602
3rd year	1.2 (0.5 to 3.0)	0.688
4th year	1.4 (0.5 to 3.5)	0.512
5th year	0.6 (0.2 to 2.1)	0.469
6th year	0.8 (0.3 to 2.3)	0.704
Financial situation		
Not enough money	base	
Sufficient money	0.9 (0.3 to 2.6)	0.824
Surplus money	1.0 (0.3 to 3.0)	0.958
Family history (parents/siblings) of chronic disease		
No	base	
Yes	3.3 (1.9 to 5.7)	<0.001

## Data Availability

The raw data supporting the conclusions of this article will be made available by the authors on request.
